# The Relationship Between the Gut Microbiome and the Aqua Cultured Fish, General Prospects Toward Fish Health: A Systematic Review

**DOI:** 10.1007/s12010-025-05330-0

**Published:** 2025-07-22

**Authors:** Darren Dean Tay, Vijay Subbiah Kumar, Rossita Shapawi, Muhammad Dawood Shah, Hajar Fauzan Ahmad, Nurzafirah Mazlan

**Affiliations:** 1 Higher Institution Centres of Excellence, Borneo Marine Research Institute, Universiti Malaysia Sabah, 88400 Kota Kinabalu, Sabah Malaysia; 2 Biotechnology Research Institute, University Malaysia Sabah, 88400 Kota Kinabalu, Sabah Malaysia; 3 Faculty of Industrial Sciences and Technology, Universiti Malaysia Pahang Al-Sultan Abdullah, 26300 Kuantan, Pahang Malaysia

**Keywords:** Marine finfish, Aquaculture, Gut microbiome, Seafood security

## Abstract

Aquaculture allows the cultivation of aquatic life outside its normal origins which can provide work opportunities, seafood security, as well as conservation efforts for endangered fish species. Numerous factors influence the health of aquaculture fish, with the gut microbiome playing a pivotal role. Research indicates that an imbalance or dysbiosis in the gut microbiome can significantly affect the overall well-being and health outcome of these fish. Despite extensive research utilizing metagenomics across diverse environments and controlled conditions, a clear consensus on the characteristic of “healthy” or “optimal” gut microbiome in domesticated fish has yet to be established. This review will cover 28 studies, which further discusses the findings of the gut microbiome within fish and attempts to provide a general outline of how the gut bacteria may interact and affect fish health within aquaculture environments. The indices as well as pathogens and beneficial bacteria of each study are also listed. This review aims to provide readers with an enhanced understanding of the complex dynamics of the gut microbiome in aquaculture fish, while offering insights that could inform the design of future studies in this field.

## Introduction

Aquaculture’s contribution to society is vast, allowing the cultivation of aquatic life outside its original environment. The process can be defined as the practice of breeding, raising, and harvesting aquatic life under very specific conditions [10]. There are multiple benefits in which aquaculture provide. Among these include food security and nutrition, providing a reliable source of protein and nutrients to the public, economic benefits, creating job opportunity and market interest; environmentally sustainable; helps reduce pressure on overfished wild stock and restore aquatic ecosystems; as well as conservation and biodiversity efforts, reducing the need to capture wild fish and potentially improving the status of endangered species [45]. Fish tend to adapt to their environment and as such their gut bacteria or gut microbiome can easily be affected by their living conditions.

The gut microbiome refers to the collective group of microorganisms within the gut which function in a complex network of interactions toward their host [4]. These complex microbial communities function to maintain the overall health and well-being of the fish host. Studies showed that these intricate relationships can aid the host through improving their nutrient absorption, enhancing their overall growth, and modulating their immune responses as well as providing protection against pathogens [18]. Conversely, the gut microbiome may also harm the host since an imbalance or dysbiosis state can increase the likelihood of disease progression in said fish [76].

Given its suggested role in fish health, it is reasonable to assume that a definitive healthy baseline exists for most fishes. This information may prove useful to farmers in their aquaculture efforts to better care for their fish. Such knowledge may help individuals to recognize potential health risks their fish may face or better formulate feeds that can improve their health and growth. Furthermore, gut microbes are reflective of the host’s environment and diet, potentially enabling farmers to gauge how well adjusted their fish are to the captive aquaculture environment.

This systematic review aims to give readers an overall understanding of how the gut microbiome can affect fish being kept in captivity for aquaculture purposes, as well as provide a general pattern of gut microbiota profile in said fish. This is done by comparing the significant findings of studies which focuses on the gut microbiome within fish being kept in various conditions. In addition, this systemic review also provides a summary of the indices used in analyzing the diversity pattern of gut microbes found within aquaculture fish and their potential impact within each respective study. It is hoped that the findings of this review may help others better understand the relationship of the gut microbiome in aquaculture fish health. This review may also prove useful for farmers moving forward in designing better and potentially cheaper healthcare strategies within the aquaculture sector.

## Material and Methods

### Data Sources and Search Strategy

The Preferred Reporting Items for Systematic Revies and Meta-Analysis (PRISMA) 2020 guidelines were used to guide in reporting this systematic review [49]. Primary articles were retrieved from Science Direct, Scopus, PubMed, and Web of Science databases. The articles were retrieved from their inception up to August 2024. The following string of search terms: gut microbiome, gut metabolome, gut microbiota, fish, were used for the screening process. All search results were filtered by language (English only). Articles that were published prior to the year 2020 were not included in the screening. Article information such as title and abstract were exported into a Microsoft Excel (Version 2410, Build 16.0.18129.20100) for quick parsing pre-evaluation process. Duplicate records were removed accordingly. After pre-evaluation, articles were downloaded based on availability.

### Study Selection and Data Extraction

Article entries were first parsed and filtered based on abstract and title. Only entries which contained gut and microbiome were maintained. Entries which mentioned or use other animal subject besides aquatic life were removed. Articles were further filtered to remove any entries that did not focus on fish for the study. DDT independently screened remaining entries for eligibility based on selection criteria before evaluating full texts. Any disagreements or discrepancies were discussed with NBM and were resolved by consensus. Entries were also excluded if they were no full texts available, a review, or non-English articles were included.

### Quality Assessment

The Mixed Methods Assessment Tool (MMAT) [44] was used to appraise the quality of included studies. The overall quality assessment of studies was evaluated independently by DDT and NBM. Based on each aspect of the quality assessment, the overall appraisal results were interpreted as the lowest score of the study components that met the criteria. All results of the study were presented in tables and figures using narrative format.

## Results

### Literature Search for Gut Microbiome in Fish Studies

**Fig. 1:**
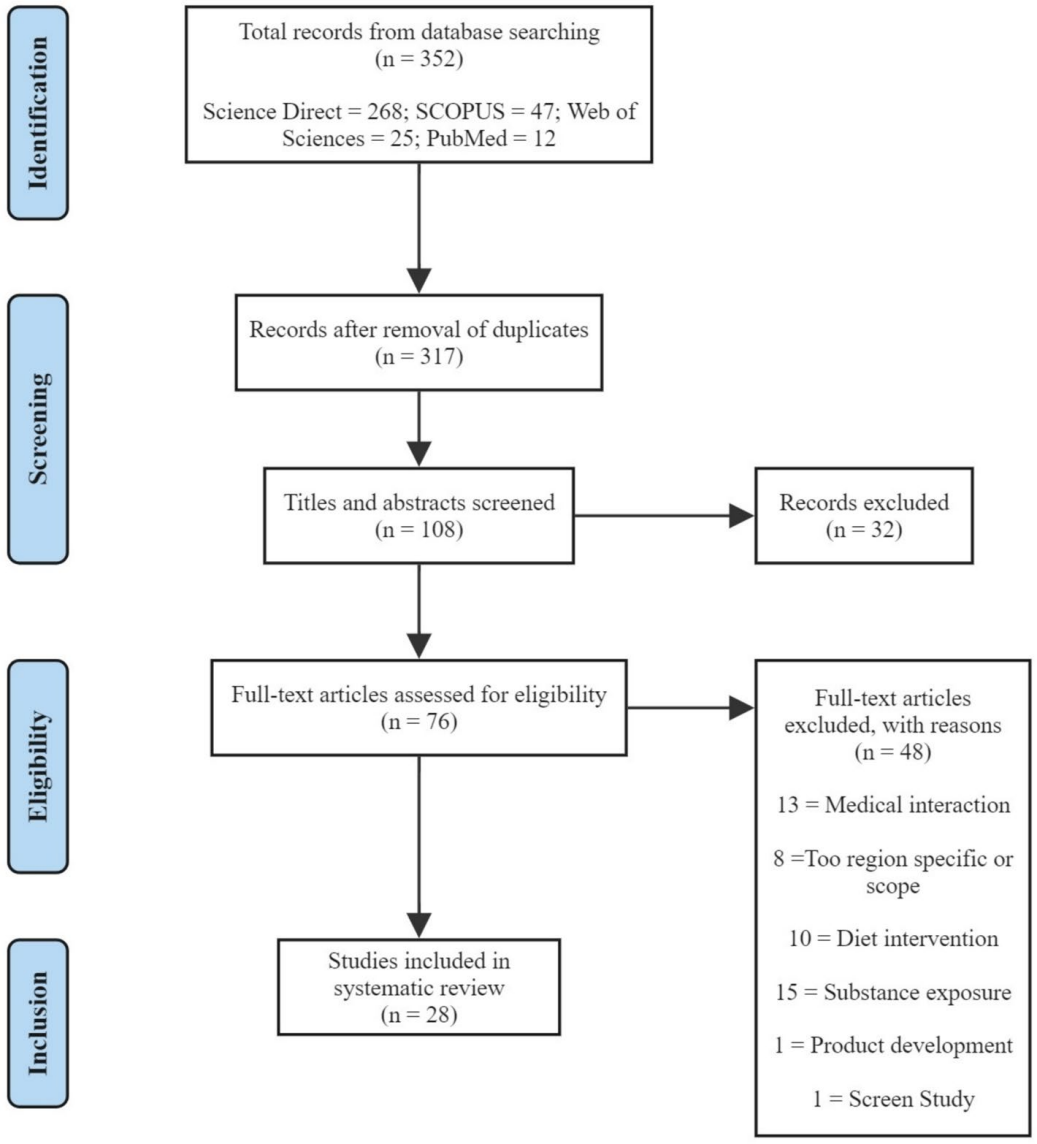
PRISMA flowchart of literature search and inclusion process

In total, 352 articles were found through the combined database search. After the removal of duplicate entries (*n* = 317), abstracts and titles which were not aquatic-based (*n* = 128) were excluded. Reports were then further filtered to only include fish-based studies (*n* = 108). A total of 108 studies were screened based on tittles and abstract. A total of 76 full text articles were reviewed for eligibility. Finally, 28 studies were included for this systematic review (Fig. 1).

### Study Characteristic

Details regarding studies which met the inclusion criteria are presented in Table 1. Compulsory characteristics of the studies to be included for review were (1) case–control studies of gut microbiomes for fish samples, (2) DNA samples which were isolated at least from fish guts, (3) included a method for gut microbiome characterization and quantification, (4) utilized next-generation sequencing (NGS) which targeted either V3 or V4 or both V3 and V4 regions of the 16S rRNA gene, (5) is gut microbiome-focused and reported contributions of gut microbiome in fish health through alpha- and beta-diversity metrics assessment, and (6) fish specimens were kept in tanks or sea cages. Studies which involved other factors that do not focus on gut microbiome involvement toward fish breeding or raising environments, such as usage of specific feed formulations, testing of treatment methods, responses to medical conditions or stimulus, and are too specific in region location or scope, were excluded from the review.

**Table 1:** Summary data about review studies predicated on sample characteristics, methodological approaches, and levels of diversity

Study (author, year)	Origin	Species	Sample size		Purpose	Sample source	Methodology	Alpha-diversity metrics	Beta-diversity metrics
			Treatment	Control					
Kong et al. [30]	Hainan, China	*Trachinotus ovatus*	Antibiotic treated *N* = 6	Control group *N* = 6	Investigated the role of gut microbiota in improving growth performance of *T. ovatus*	Intestines	Sequencing targeted at 16S rDNA gene from V4 region	Shannon ACE Faith’s PD	Bray–Curtis
Balasubramanian et al. [7]	Coimbatore, Tamil Nadu, India	*Danio rerio*	Treated *N* = 30	Control *N* = 30	Investigated the effects of copper oxide nanoparticles (CuO NPs) on the gut microbiome of *D. rerio*	Intestines	Sequencing targeted at 16S rRNA gene from V3–V4 region	Shannon Simpson Observed OTU Chao1	Bray–Curtis Unweighted-UniFrac Weighted-UniFrac Jaccard distance
Choi et al. [16]	Jegok-gil, Jinju, South Korea	*Danio rerio*	Treatment *N* = 10 (PP: *n* = 5) (UV-PP, *n* = 5)	Control *N* = 5	Investigated the effects of polypropylene microplastics exposure towards the gut microbiome of *D. rerio*	Intestines	MiSeq sequencing targeting 16S rRNA gene from V3–V4 region	Chao1 Shannon	Bray Curtis Jensen-Shannon
Liu et al. [36, 38]	Liaoning, China	*Takifugu rubripes*	Treatment *N* = 18 (50 µg: *n* = 6) (100 µg: *n* = 6) (500 µg: *n* = 6)	Control *N* = 6	Investigated the effects of copper exposure towards the gut microbiome of *T. rubripes*	Intestines	Novaseq sequencing targeting the 16S rRNA gene from the V4 region	Shannon Simpson	Unweighted UniFrac
Zhao et al. [79]	Qingdao, China	*Danio rerio*	Treatment *N* = 16 (1 µg: *n* = 6) (100 µg: *n* = 6) *M:F even dist*	Control *N* = 6 (Male = 3) (Female = 3)	Investigated the effects of florfenicol exposure towards gut microbiome of male and female *D. rerio*	Intestines	NovaSeq6000 sequencing targeting the 16S rRNA gene from V3–V4 region	Observed OTU Chao1 Shannon	Unidentified PCoA
Byeon et al. [11]	Suwon, South Korea	*Oryzias melastigma*	Treatment *N* = 5	Control *N* = 1	Investigated the effects of phenanthrene and microplastics towards the gut microbiome of male *O. melastigma*	Intestines	MiSeq sequencing targeting the 16S rRNA gene from V3–V4 region	Observed OTU Shannon Chao1	Unspecified PCoA
Wang et al. [67]	Wuhan, China	*Danio rerio*	Treatment *N* = 16 (TCPP: 4 M,4F) (RDP: 4 M,4F)	Control *N* = 8 (4 male, 4 female)	Investigated the effects of diphenyl phosphate exposure towards the gut microbiome of both male and female *D. rerio*	Intestines	MiSeq PE300 sequencing targeting the 16S rRNA gene from V3–V4 region	Shannon ACE Simpson Chao1	Bray–Curtis
Liu et al. [36, 38]	Yangzhong, China	*Coilia* *nasus*	Treatment *N* = 6 (AE: 3) (AES: 3)	Control *N* = 3	Investigated the effects air exposure stress towards the gut microbiome of *C. nasus* and its mitigation through salinity concentration	Intestines	Metagenome sequencing through ONT systems	ACE Chao1 Shannon Simpson	Unspecified PCoA
Zhu et al. [83]	Wuhan, China	*Danio rerio*	Treatment *N* = 3	Control *N* = 3	Investigated the effects of polystyrene nanoplastic exposure towards the gut microbiome of *D. rerio*	Intestines	MiSeq sequencing targeting the 16S rRNA gene	Simpson	Unspecified PCoA
Yu et al. [75]	Yangling, China	*Micropterus salmoides*	Treatment *N* = 9 (SC: 3) (LC: 3) (SH: 3)	Control *N* = 3	Investigated the effects of acute temperature stresses toward the gut microbiome of *M. salmoides*	Intestines	HiSeq 4000 sequencing targeting the 16S rRNA gene from V3–V4 region	ACE Chao1 Shannon Simpson	Unweighted UniFrac (NMDS)
Zhou et al. [81]	Linxia, China	*Oncorhync-hus mykiss*	Treatment *N* = 6	Control *N* = 6	Investigated the effects of heat stress exposure toward the gut microbiome of *O. mykiss*	Intestines	HiSeq 2500 sequencing targeting the 16S rRNA gene from the V3–V4 region	ACE Chao1 Shannon Simpson	Bray–Curtis Jaccard/NMDS
Chen et al. [14]	Jiangsu, China	*Carassius auratus gibelio*	Treatment *N* = 3	Control *N* = 3	Investigated how the gut microbiome of *C.a. gibelio* changes from being exposed to CyHV-2 infection	Intestines	MiSeq sequencing targeting the 16S rRNA gene from the V4 region	Chao1 Simpson Shannon	PCoA/weighted UniFrac
Zhang et al. [78]	Xinxiang, China	*Cyprinus carpio*	Treatment *N* = 6	Control *N* = 6	Investigated the effects of *Aeromonas hydrophila* infection toward gut microbiome changes in *C. carpio*	Intestines	MiSeq sequencing targeting the 16S rRNA gene from the V3–V4 region	Chao1 Simpson Shannon	Unweighted UniFrac
Bao et al. [9]	Hainan, China	*Oreochromis mossambicus*	Treatment *N* = 40 (assumption) (PLA: 20) (PVC: 20)	Control *N* = 20 (assumption)	Studied the effects of biodegradable and conventional microplastics in the gut microbiome of *O. mossambicus*	Gut	MiSeq sequencing targeting the 16S rRNA gene from the V3–V4 region	Chao1 Shannon	Analysis through PERMANOVA
Fang et al. [21]	Gansu, China	*Brachymystax lenok tsinlingensis Li*	Treatment *N* = 3	Control *N* = 3	Studied the effects of high temperature stress towards the gut microbiome within *B.l.l.Li* fish	Gut	Unspecified sequencing targeting the 16S rRNA gene	ACE Simpson Shannon	NMDS PCoA
Nie et al. [46]	Yunnan, China (*)	*Cyprinus* *carpio*	Study groups *N* = 50 (SG: 21) (FG: 29)	No control group	Investigated the relationship between the growth rate of *C. carpio* and their gut microbiome	Gut	MiSeq sequencing targeting the 16S rRNA gene from the V3–V4 region	ACE Shannon Simpson Good coverage	Bray–Curtis
Liao et al. [34]	Xiamen, China	*Oryzais* *melastigma*	Treatment *N* = 36 (NC: 12) (PS: 12) (MT: 12)	Control *N* = 12	Studied the joint effects of microplastics and tetracycline on the gut microbiome of *O. melastigma*	Gut	HiSeq 4000 sequencing targeting the 16S rRNA gene from the V4–V5 region	None reported	Bray–Curtis
Ye et al. [74]	China	*Hypophthalmichthys nobilis*	Treatment *N* = 30 (NC:10) (PY:10) (XHK:10)	No defined Control group	Researched on how different culture system can impact the gut microbiome of *H. nobilis*	Gut	NovaSeq 6000 sequencing targeting the 16S rRNA gene from the V3–V4 region	Shannon Simpson Chao1 ACE	Bray–Curtis
Medriano & Bae [43]	Singapore	*Danio rerio*	Treatment *N* = 12 Polyester: 6 Polyethylene: 6	Control *N* = 6	Investigated the effects of acute microplastics exposures towards the gut microbiome of *D. rerio*	Gut	MiSeq sequencing targeting the 16S rRNA gene from the V3–V4 region	Observed OTU Faith’s PD Pielou’s Evenness	Unweighted Unifrac distance
Lu et al. [39]	Wuhan, China	*Oreochromis niloticus*	Treatment *N* = 12 (27 μm_4%: 4) (27 μm_8%: 4) (63 μm_4%: 4) (63 μm_8%: 4)	Control *N* = 3	Comprehensive study understanding impacts of dietary exposure of polyethylene microplastics on the gut microbiome of farmed *O. niloticus*	Gut	MiSeq sequencing targeting the 16S rRNA gene from the V4 region	Shannon Simpson	No reported measurement use
Jia et al. [25]	Shanghai, China	*Ctenopharyngodon idella*	Treatment *N* = 14 (100 μg/L: 7) (1000 μg/L: 7)	Control *N* = 7	Investigated the effects microplastic exposure towards the altered gut microbiome of *C. idella*	Gut	Sequencing targeted at 16S rRNA gene from V3–V4 region	Shannon Simpson	Bray–Curtis
Hou et al. [24]	Wuhan, China	*Gobiocypris rarus*	Treatment *N* = 3	Control *N* = 3	Studied the effects of long-term exposure to microplastics within the gut microbiome of *G. rarus*	Gut	MiSeq sequencing targeting the 16S rRNA gene from the V3–V4 region	Shannon	No reported measurement use
Chen et al. [13]	Xinxiang, China	*Cyprinus carpio L*	Treatment *N* = 48 6 per group: GLY-L, GLY-H, MPs-L, MPs-H, GLY-L + MPs-L, GLY-L + MPs-H, GLY-H + MPs-L, GLY-H + MPs-H	Control *N* = 6	Studied the long-term exposure of polyethylene microplastics and glyphosate towards the gut microbiome of *C. carpio L*	Gut	MiSeq sequencing targeting the 16S rRNA gene from the V3–V4 region	Chao1 Shannon	Weighted UniFrac distance
Usman et al. [64]	Selangor, Malaysia	*Oryzias javanicus*	Treatment *N* = 12	Control *N* = 4	Investigated the effects of polystyrene microplastics on the gut microbiome of *O. javanicus*	Fecal sample	MiSeq sequencing targeting the 16S rRNA gene from the V3–V4 region	Simpson Shannon	No reported measurement use (Relies on other)
Liu et al. [37]	Dalian, China	*Dicentrarchus labrax*	Treatment *N* = 18 T1: 9 T3: 9	Control *N* = 9	Studied how the gut microbiome of *D. labrax* is affected by temperature stress	Gut	NovaSeq 6000 sequencing targeting the 16S rRNA gene from the V4 region	Observed OTU Chao1 Simpson ACE Goods Coverage Faith’s PD	Weighted Unifrac Unweighted Unifrac
Guo et al. [22]	Yantai, China	*Danio rerio*	Treatment *N* = 20 4 per group: ENR, CMPs, RMPs, ECMPs, ERMPs	Control *N* = 4	Investigated the toxic effects of commercial and realistic polystyrene microplastics on the gut microbiome of *D. rerio*	Gut	NovaSeq sequencing targeting the 16S rRNA gene from the V4 region	Chao1 Simpson	Bray–Curtis
Zhao et al. [80]	Hangzhou, China	*Danio rerio*	Treatment *N* = 18 6 parallel samples per group: 10,100, and 1000 mg/L PE-MPs	Control *N* = 6 parallel samples	Studied the effects of polyethylene microplastics on the microbiome of larval *D. rerio*	Evenly crushed zebrafish (tissue)	NovaSeq sequencing targeting the 16S rRNA gene from the V3–V4 region	Shannon Simpson	Bray–Curtis
Ouyang et al. [47]	Shanghai, China	*Cyprinus carpio*	Treatment *N* = 24 MP100-1, MP1000-1	Control *N* = 12	Investigated the effects of microplastics intake within the gut microbiome of *C. carpio*	Gut	MiSeq sequencing targeting the 16S rRNA gene from the V3–V4 region	Simpson Chao1	Weighted UniFrac

The results of appraising the studies based on MMAT can be found in Table 2. The overall qualities of the 28 non-randomized studies were classified high-quality studies as the overall score ranged from 60 to 100%. Nevertheless, the intervention administered, or exposure of the occurred methodology quality criteria, was not relevant to the research interest of the review hence were labeled as not applicable (NA).

**Table 2:** Quality assessment using the Mixed Method Appraisal Tool (MMAT) for quantitative non-randomized studies

First author (year)	Type of study	Screening questions		Methodology quality criteria					Overall quality score (%)
		Are there clear research questions?	Do the collected data allow to address the research questions?	Are the participants representative of the target population?	Are measurements appropriate regarding both the outcome and intervention (or exposure)?	Are there complete outcome data?	Are the confounders accounted for in the design and analysis?	During the study period, is the intervention administered (or exposure occurred) as intended?	
Kong et al. [30]	Case–control study	100%	100%	80%	100%	100%	100%	NA	80%
Balasubramanian et al. [7]	Case–control study	100%	100%	100%	100%	100%	100%	NA	100%
Choi et al. [16]	Case–control study	100%	100%	80%	100%	100%	100%	NA	80%
Liu et al. [36, 38]	Case–control study	100%	100%	100%	100%	100%	100%	NA	100%
Zhao et al. [79]	Case–control study	100%	100%	100%	100%	100%	100%	NA	100%
Byeon et al. [11]	Case–control study	100%	100%	80%	100%	100%	100%	NA	80%
Wang et al. [67]	Case–control study	100%	100%	100%	100%	100%	100%	NA	100%
Liu et al. [36, 38]	Case–control study	100%	100%	100%	100%	100%	100%	NA	100%
Zhu et al. [83]	Case–control study	100%	100%	80%	80%	100%	100%	NA	80%
Yu et al. [75]	Case–control study	100%	100%	100%	100%	100%	100%	NA	100%
Zhou et al. [81]	Case–control study	100%	100%	100%	100%	100%	100%	NA	100%
Chen et al. [14]	Case–control study	100%	100%	100%	100%	80%	100%	NA	80%
Zhang et al. [78]	Case–control study	100%	100%	100%	100%	100%	100%	NA	100%
Bao et al. [9]	Case–control study	100%	100%	100%	100%	80%	100%	NA	80%
Fang et al. [21]	Case–control study	100%	100%	100%	100%	80%	100%	NA	80%
Nie et al. [46]	Preliminary study	100%	100%	100%	100%	100%	100%	NA	100%
Liao et al. [34]	Case–control study	100%	100%	100%	100%	60%	100%	NA	60%
Ye et al. [74]	Preliminary study	100%	100%	100%	100%	100%	100%	NA	100%
Medriano and Bae [43]	Case–control study	100%	100%	100%	100%	100%	100%	NA	100%
Zhang et al. [78]	Case–control study	100%	100%	100%	100%	60%	100%	NA	60%
Jia et al. [25]	Case–control study	100%	100%	100%	100%	80%	100%	NA	100%
Hou et al. [24]	Case–control study	100%	100%	100%	100%	60%	100%	NA	60%
Chen et al. [13]	Case–control study	100%	100%	100%	100%	100%	100%	NA	100%
Usman et al. [64]	Case–control study	100%	100%	100%	100%	60%	100%	NA	60%
Liu et al. [37]	Case–control study	100%	100%	100%	100%	100%	100%	NA	100%
Guo et al. [22]	Case–control study	100%	100%	100%	100%	100%	100%	NA	100%
Zhao et al. [80]	Case–control study	100%	100%	100%	100%	100%	100%	NA	100%
[47]	Case–control study	100%	100%	100%	100%	100%	100%	NA	100%

•The score was presented as 100% quality criteria met; 80% quality criteria met; 60% quality criteria met; 40% quality criteria met; 20% quality criteria met.

### Diversity Analysis of the Gut Microbiome Within Domesticated Fish

All 28 of the included studies utilized the 16S rRNA gene to sequence the gut microbiome of fish in their respective studies. There was one exception to this which was the study by Liu et al. [36, 38] which utilized metagenomic sequencing through ONT systems. All studies also made use of alpha- and beta-diversity metrics in some manner to report on the significance of gut microbiome toward their studies. While there were studies which did not completely disclose the diversity metrics used, only the studies under Liao et al. [34] and Hou et al. [24] did accurately report or shared their data completely. Regardless, these studies still reported their diversity findings using less-standardized methods.

**Fig. 2:**
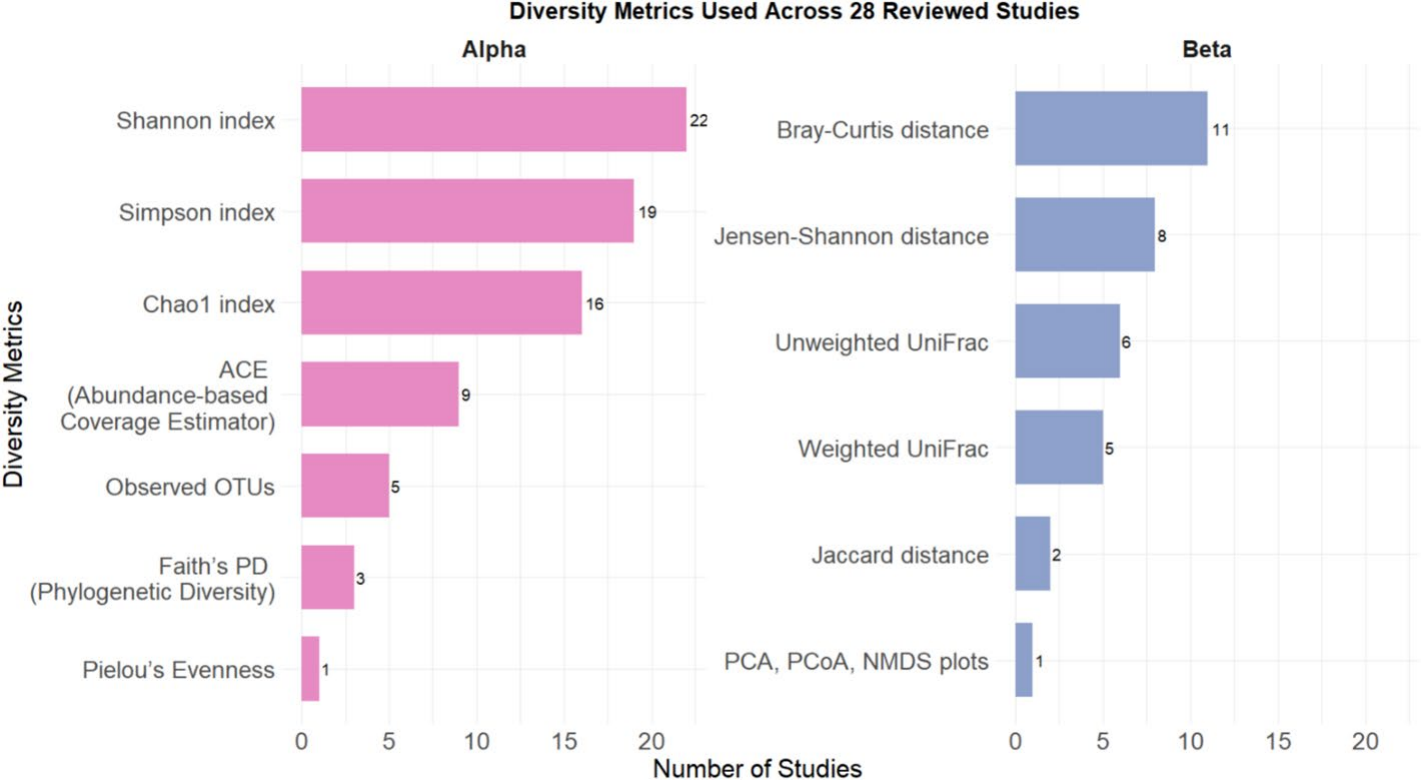
Frequency of alpha- and beta-diversity metrics used across reviewed studies. Shannon and Bray–Curtis were the most utilized metrics, with great variation for metric selection between studies

The results of the diversity findings for each study are recorded in Table 3. For alpha-diversity, metrics that were employed included Shannon, Simpson, Ace, Faith’s phylogenetic diversity, observed OTUs, Chao1, and Pielou’s indices. All studies implored the use diversity metrics which accounted for both species richness and evenness except for the two earlier mentioned studies. For beta diversity, the diversity metrics of Bray Curtis, Jaccard, unweighted Unifrac, weighted Unifrac, and Jensen-Shannon distances were utilized. The most common metrics utilized were Shannon index (*n* = 19), followed by Chao1 and Simpson for alpha-diversity, whereas Bray–Curtis distance (*n* = 11) was the most popular option for beta-diversity (Fig. 2). Many studies however did not include their statistical data but still presented PCA plots of their findings. Overall, the alpha-diversity was significant for roughly half of the included studies, whereas beta-diversity was mostly found to be significant across the studies.

**Table 3:** Statistical analysis of diversity assessment of reviewed studies

First author (year)	Alpha-diversity		Beta-diversity	
Kong et al. [30]	Significant alpha diversity results where species richness was higher within control group vs. antibiotic treated group	*Shannon index (p* < *0.01)* *Ace index (p* < *0.01)* *Faith’s PD (p* < *0.01)*	Composition was reported to be different between study groups. Results were supplemented with LEfSe results where certain features contributed to differences between groups	NA
Balasubramanian et al. [7]	Overall, alpha diversity was reduced within treated groups but there was no statistical significance in species richness and evenness	*Shannon index (p ˃ 0.05)* *Simpson index (p ˃ 0.05)* *Observed OTU (p ˃ 0.05)* *Chao1 index (p ˃ 0.05)*	No significant differences in composition or community structure across study groups	*Bray Curtis (p ˃ 0.05)* *Jaccard distance (p ˃ 0.05)* *Unweighted Unifrac distance* *(p ˃ 0.05)* *Weighted Unifrac distance* *(p ˃ 0.05)*
Choi et al. [16]	Alpha diversity was overall higher in exposed groups compared to control. Differences were significant	*Chao1 index (p* < *0.05)* *Shannon index (p* < *0.05)*	Samples were observed to not cluster together, suggesting different compositions	*Bray Curtis (p* < *0.05)* *Jensen-Shannon (p* < *0.05)*
Liu et al. [36, 38]	Alpha diversity was found lower in exposed groups, but no significant differences were found	*Shannon index (p ˃ 0.05)* *Simpson index (p ˃ 0.05)*	Gut microbiome composition was found significantly different across study groups	*Unweighted Unifrac distance (p* < *0.05)*
Zhao et al. [79]	No statistical differences for observed or Chao1 metrics, but Shannon showed significant differences for male study	*Observed OTU (p* = *0.099)* *Chao1 index (p* = *0.29)* *Shannon index (p* = *0.027)*	Male study showed distanced clustering of samples, indicating different microbial composition	NA
	No statistical differences for observed or Chao1 metrics, but Shannon showed significant differences for female study	*Observed OTU (p* = *0.67)* *Chao1 index (p* = *0.73)* *Shannon index (p* = *0.027)*	Female study showed distanced clustering of samples, indicating different microbial composition	NA
Byeon et al. [11]	Alpha diversity for Shannon index was reported to significantly different across study groups	*Observed OTU (p ˃ 0.05)* *Shannon index (p* < *0.05)* *Chao1 index (p ˃ 0.05)*	Beta-diversity was reported to be significantly different across study groups, except for the control and Phe + MP_L group	NA
Wang et al. [67]	Male RDP-exposed fish showed significantly higher diversity than control	*Shannon index (p* = *0.0341)* *ACE index (p* = *0.0106)* *Simpson index (p ˃ 0.05)* *Chao1 index (p* = *0.0083)*	Male RDP-exposed group showed significant difference in microbial composition	*Bray Curtis (p* = *0.029)*
	Females showed significantly lowered alpha-diversity for ACE index	*Shannon index (p ˃ 0.05)* *ACE index (p* < *0.05)* *Simpson index (p ˃ 0.05)* *Chao1 index (p ˃ 0.05)*	Females showed no significant differences in terms of microbial composition	*Bray Curtis (p ˃ 0.05)*
Liu et al. [36, 38]	There was no significant difference in terms of alpha-diversity across study groups	*ACE index (p ˃ 0.05)* *Chao1 index (p ˃ 0.05)* *Shannon index (p ˃ 0.05)* *Simpson index (p ˃ 0.05)*	PCoA plots showed clear separations across groups, signifying different microbial compositions	NA
Zhu et al. [83]	Species diversity was significantly lowered in exposed group	*Simpson index (p* = *0.037)*	Study groups showed distanced clustering, indicating different microbial composition	NA
Yu et al. [75]	No significant differences at 24-h mark for all metrics	*ACE index (p ˃ 0.05)* *Chao1 index (p ˃ 0.05)* *Shannon index (p ˃ 0.05)* *Simpson index (p ˃ 0.05)*	Microbial composition was reported to be different across study groups	NA
	Lowered diversity within SH but Simpson index was significantly different within CK group after 48-h mark	*ACE index (p ˃ 0.05)* *Chao1 index (p ˃ 0.05)* *Simpson index (p* < *0.05)*		
Zhou et al. [81]	Overall results for alpha diversity were found to be not significant in the study. Diversity was not affected	*ACE index (p ˃ 0.05)* *Chao1 index (p* < *0.05)* *Shannon index (p ˃ 0.05)* *Simpson index (p ˃ 0.05)*	PCoA plots showed separations between study groups, indicating altered gut microbiome compositions. However, there was no significant differences	*Bray Curtis (p* = *0.10)*
Chen et al. [14]	Shannon index was reported to be lower in control while Simpson was higher. There were no significant differences in bacterial diversity though	*Chao1 index (p ˃ 0.05)* *Simpson index (p ˃ 0.05)* *Shannon index (p ˃ 0.05)*	PCoA plots showed clear separation between study group, indicating significantly different gut microbiome composition	NA
Zhang et al. [78]	Overall results showed that species diversity was lowered but no statistical differences were found	*Chao1 index (p ˃ 0.05)* *Simpson index (p ˃ 0.05)* *Shannon index (p ˃ 0.05)*	Plots were observed to be separated between study groups, implying different microbial compositions	NA
Bao et al. [9]	Species diversity and abundance was reported lower in treated group, however no significant differences were found	*Chao1 index (p ˃ 0.05)* *Shannon index (p ˃ 0.05)*	PCA results showed distinct separation between groups, microbial composition was significantly different between study groups	*PCA (PERMANOVA) (p* = *0.0022)*
Fang et al. [21]	ACE index reported lower diversity with increased temperature, though no statistical significance was found. Both Simpson and Shannon index were found to be significantly higher at increased temperature	*ACE index (p* = *0.27)* *Simpson index (p* = *0.004)* *Shannon index (p* = *0.001)*	Both PCA and PCoA plots showed distinct groups forming across samples, indicating significant differences in microbial composition between study groups	NA
Nie et al. [46]	Alpha diversity revealed that richness and diversity was significantly higher within SG group compared to FG group	*ACE index (p* < *0.05)* *Shannon index (p* < *0.05)* *Simpson index (p* < *0.05)*	Beta-diversity was significant, microbial composition is different between SG and FG group	*Bray Curtis (p* = *0.029)*
Liao et al. [34]	No reported uses of alpha-diversity indices	NA	NMDS showed separate cluster formation, hinting possible differences in composition	NA
Ye et al. [74]	No significant differences were found for alpha-diversity	*Shannon index (p* = *0.063)* *Simpson index (p* = *0.07)* *Chao1 index (p* = *0.093)* *ACE index (p* = *0.115)*	Beta-diversity was significant, microbial composition was different between study groups	*Bray Curtis (p* = *0.001)*
Medriano & Bae [43]	Based on observed OTU, microbial richness was lowered in exposed group but overall alpha-diversity was still considered to be not significant	*Observed OTU (p* = *0.001)* *Faith’s PD (p* < *0.05)* *Shannon index (p* < *0.05)* *Pielou index (p* < *0.05)*	Microbial composition was significantly different between study groups	*Unweighted Unifrac distance (p* < *0.05)*
Lu et al. [39]	Alpha-diversity was significant for 63 μm_8% group	*Shannon index (p* < *0.05)* *Simpson index (p* < *0.05)*	No reported measurement use of beta-diversity indices	NA
Jia et al. [25]	MPs-H group was significantly lowest species diversity and richness versus other groups	*Shannon index (p* < *0.05)* *Simpson index (p* < *0.05)*	Significantly different microbial composition between study groups	*Bray Curtis, (p* = *0.001)*
Hou et al. [24]	Microbial diversity was reported to increase within exposed PS-MPs groups but no significant statistical was found	NA	No reported usage of beta-diversity indices but PLS-DA plot showed clear separation of study groups, indicating possibility of differences in composition	NA
Chen et al. [13]	Alpha-diversity was found to be significant for selected MP groups	*Chao1 index (p* < *0.05)* *Shannon index (p* < *0.05)*	Microbial composition was significantly difference between study groups	*Weighted UniFrac distance* *(p* = *0.001)*
Usman et al. [64]	Species richness and diversity was significantly different between study groups	*Simpson index (p* < *0.05)* *Shannon index (p* < *0.05)*	PCA plot showed clear distancing of groups between each other, signifying different compositions	NA
Liu et al. [37]	No reported statistical significance for alpha-diversity	*Observed OTU (p ˃ 0.05)* *Chao1 index (p ˃ 0.05)* *Simpson index (p ˃ 0.05)* *Ace index (p ˃ 0.05)* *Faith’s PD (p ˃ 0.05)*	There was no reported statistical significance for beta-diversity	*Weighted Unifrac distance* *(p* < *0.05)* *Unweighted Unifrac distance* *(p* < *0.05)*
Guo et al. [22]	No statistical significance for Chao1 but there are statistical differences for alpha-diversity based on Simpson	*Chao1 index (p ˃ 0.05)* *Simpson index (p* < *0.01)*	Microbial composition was significantly different for all groups except for ECMP and ERMP groups	*Bray Curtis (p* < *0.05)*
Zhao et al. [80]	Alpha-diversity was reported to be significant different between groups	*Shannon index (p* < *0.05)* *Simpson index (p* < *0.05)*	Microbial composition was significantly different between groups	*Bray Curtis (p* < *0.05)*
Ouyang et al. [47]	No significant differences found for species diversity and richness between study groups	*Simpson index (p ˃ 0.05)* *Chao1 index (p ˃ 0.05)*	Plots showed clear separation, and microbial composition was significantly different between groups	*Weighted UniFrac distance (p* = *0.001)*

### Taxa Associations Within Domesticated Fish

**Fig. 3:**
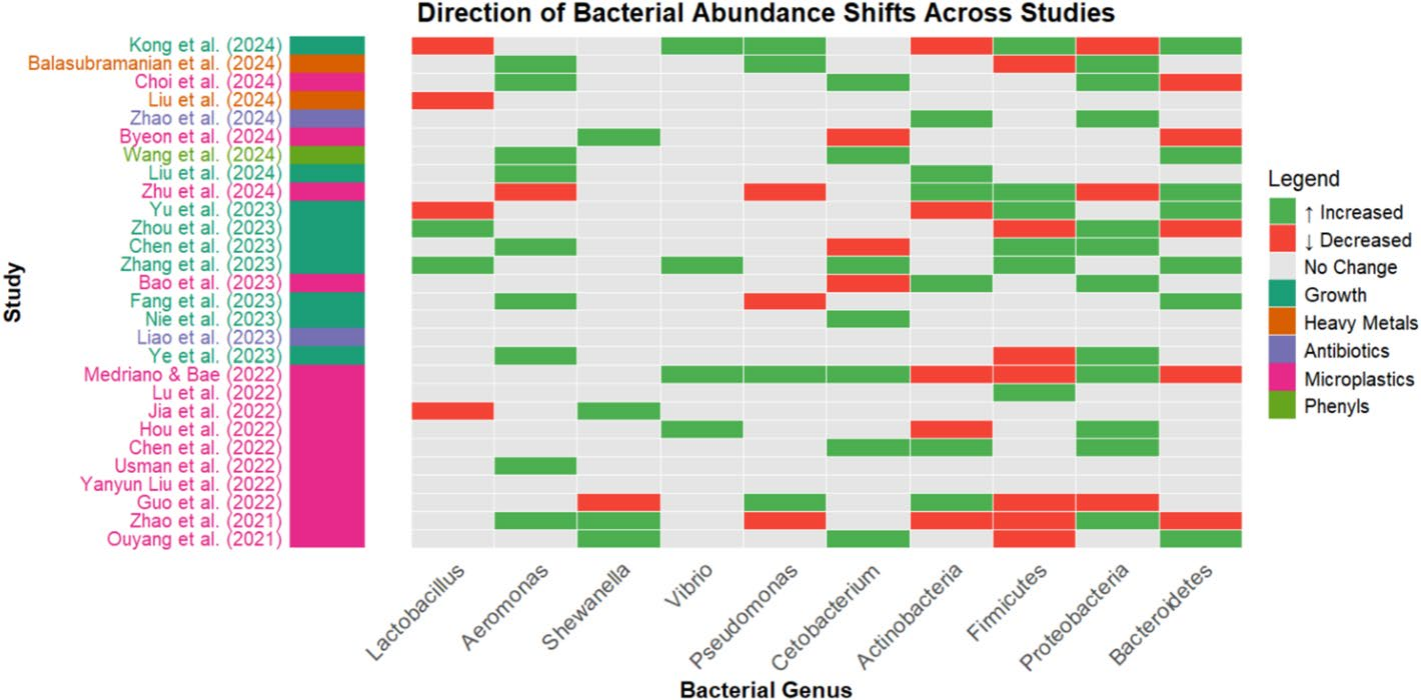
Heatmap of directional shifts in the relative abundance of reported bacteria across reviewed studies. Green tiles indicate increases, red tiles indicate decreases, and gray tiles indicate no reported changes within treatment groups in comparison to control groups. Studies are color-coded based on the area of study

The details of taxa associations in respect to the 28 studies can be found in Table 4 which contains the abundance reports, LEfSe findings, as well as reported pathogens or beneficial microbes in each study. Only microbial features at phylum and genus level were considered and reported for this review. At phylum level, the gut microbiome of most fishes comprised Proteobacteria, Firmicutes, Bacteroidetes, Actinobacteriota, and Fusobacteria. Other notable phyla include Gemmatimonadetes, Cyanobacteria, and Verrucomicrobiota. Firmicutes was the most abundant phyla followed by Proteobacteria. Across the 28 studies, numerous bacteria were reported as increasing or decreasing in abundance based on the various treatment groups (Fig. 3). Firmicutes were mostly observed to decrease within treatment groups except for the studies conducted by Yu et al. [75], Chen et al. [14], Zhang et al. [78], and Lu et al. [39]. On the other hand, most of the studies reported Proteobacteria levels to increase within treatment groups. The exception to this were the studies under Zhu et al. [83], Zhang et al. [78], and Guo et al. [22], which reported a decrease in abundance.

**Table 4:** Notable microbes found in reviewed studies

Study	Reported pathogen	Reported beneficial	LEfSe/increased abundance	Species
Kong et al. [30]	*Vibrio*,* Pseudomonas*	*Lactobacillus*	Within TB group: *Achromobacter*, *Vibrio*, *Pedobacter* and *Pseudomonas*. Higher Bacteroidota/Firmicutes but less Proteobacteria Within CB (control) group: *Saccharopolyspora*, *Lactobacillus, Sphingobium,* *Ruegeria, Rhodococcus*, and *Paracoccus.* More Actinobacteriota	*Trachinotus ovatus*
Balasubramanian et al. [7]	*Aeromonas*,* Pseudomonas*	No highlight or direct report of importance	Treatment groups: Increased Proteobacteria but decreased Firmicutes. Significant increase in *Arsenicibacter*, Flavobacteriales, *Aeromonas*, and *Pseudomonas*	*Danio rerio*
Choi et al. [16]	*Aeromonas*, *Rhizobium*, and* Gemmobacter*	*Luteolibacter*, *GQ360021-g*, *Rhodobacter*	Exposure groups: Higher levels of Fusobacteria, Proteobacteria and lowered Bacteroidetes. Increased *Cetobacterium*, *Plesiomonas*, *GQ360021-g*, *Aeromonas*, *Rhizobium*, *Gemmobacter*, *Cloacibacterium*, *Luteolibacter*, and *Rhodobacter*	*Danio rerio*
Liu et al. [36, 38]	*Staphylococcus*, *Acetatifactor*, *Catonella*, *Actinobacillus*, and* Bergeyella*	*Butyrivibrio*, *Ruminococcus_torques_group*, and *Lactobacillus*	Treatment groups: Increase of *of Rikenella*, *Hyphomicrobium*, *Marvinbryantia*, *Bauldia*, *Lachnoclostridium*, *Bergeyella*, *Actinobacillus*, *Rothia*, *Corynebacterium*, *Megasphaera*, *Catonella*, *Aerococcus*, and Absconditabacteriales_(SR1) and Fusicatenibacter Decrease of *Butyrivibrio* and *Ruminococcus_torques_group*, *Lactobacillus*, *Clostridium, Staphylococcus, Nautella*	*Takifugu rubripes*
Zhao et al. [79]	No highlight or direct report of importance	No highlight or direct report of importance	Treatment group: Increase of Verrucomicrobiota Actinobacteriota, Proteobacteria Reduction of Fusobacteria	*Danio rerio*
Byeon et al. [11]	*Photobacterium*	*Shewanella*, *Ruegeria*	Treatment group: Increase of Planctomycetes, *Photobacterium*, *Shewanella*, *Fritschea*, *Luteolibacter*, unassigned Spongiibacteraceae, *Marimicrobium*, and *Reugeria* Reduction of Bacteroidetes, Fusobacteria, *Cetobacterium*,	*Oryzias melastigma*
Wang et al. [67]	*Aeromonas*, *Streptococcus*, *Fastidiosipila*	*Thermobacillus*	Treatment group: Increase of Fusobacteria (Females), Bacteroidota (Males), *Cetobacterium*, *Aeromonas*, *ZOR0006*, *Citrobacter, Thermobacillus* (male)*, Defluviicoccus* (male), *Streptococcus* (male), *Fastidiosipila* (male) and *Ureibacillus* (male) Decrease of Fusobacteria (Males), Bacteroidota	*Danio rerio*
Liu et al. [36, 38]	*Aeromonas*, Vibrionaceae, Enterobacterales	No highlight or direct report of importance	Treatment group: Significant presence of *Aeromonas*, *Corynebacterium*, Actinobacteriota Control group: Significant presence of Enterobacterales, Vibrionaceae,	*Coilia nasus*
Zhu et al. [83]	*Acinetobacter*, *Bacillus, Prevotella 9*, and* Roseburia*	*Pseudomonas* and *Aeromonas*	Treatment group: Significant increase of Bacteroidetes, Actinobacteriota, Firmicutes, *Bacillus*, *Prevotella 9*, *Acinetobacter* and *Roseburia* Significant decrease of Proteobacteria, *Pseudomonas*, and *Aeromonas*	*Danio rerio*
Yu et al. [75]	No highlight or direct report of importance	*Muribaculaceae* and Ruminococcaceae UCG 014	Treatment group: Increase Firmicutes, *Enterobacter*, *Bacteroides*, and *Faecalibacterium*, Decrease Actinobacteriota, *Muribaculaceae*, *Lactobacillus*, *RB41*, and Ruminococcaceae UCG 014	*Micropterus salmoides*
Zhou et al. [81]	No highlight or direct report of importance	No highlight or direct report of importance	Heat-treated group: Increase Proteobacteria, *Enterobacteriaceae*, *Lactobacillus* Decrease Firmicutes, Bacteroidetes, *Clostridium*, *Acinetobacter* Significant marker for heat stress: *Entrerobacterales*	*Oncorhync-hus mykiss*
Chen et al. [14]	*Aeromonas*	*Cetobacterium*	Infected Group: Increase of Proteobacteria, Firmicutes, and *Aeromonas* Decrease of Fusobacteria, and *Cetobacterium*	*Carassius auratus gibelio*
Zhang et al. [78]	*Vibrio*	*Cetobacterium*, *Lactobacillus*	Infected group: Increase of Firmicutes, Fusobacteria, Bacteroidetes, *Vibrio*, *Lactobacillus Cetobacterium*, and *Anaerostipes* Decrease of Proteobacteria Control group: Significant enrichment of *Tenericutes*, *Rhodobacter*	*Cyprinus carpio*
Bao et al. [9]	*Legionella*	*Cetobacterium* and *Actinobacteriota*	Treatment group: Increase of Fusobacteria, Proteobacteria, *Cetobacterium* (PVC), *Actinobacteriota*, *Romboutsia*, *Legionella*, and *Bacillus* Decrease of *Cetobacterium* (PVA)	*Oreochromis mossambicus*
Fang et al. [21]	*Aeromonas*	*Pseudomonas*	Heat treatment group: Increase of Bacteroidetes, *Aeromonas*, and *Empedobacter* Decrease of Firmicutes, Gemmatimonadetes, *Pseudomonas*, *Delftia*, and *Acinetobacter*	*Brachymystax lenok tsinlingensis Li*
Nie et al. [46]	No highlight or direct report of importance	No highlight or direct report of importance	SG (slow) group: Higher abundance of Cyanobacteria, *Streptophyta*, *Cetobacterium* FG (fast) group: Higher abundance Fusobacteria, *Streptophyta*, *Cetobacterium*, Aeromonadaceae	*Cyprinus carpio*
Liao et al. [34]	*Brevundimonas* and *Stenotrophomonas*	No highlight or direct report of importance	Treatment group: Increase of Bacteroidales, Lachnospiralesm Corynebacteriales, *Microbacterium Brevundimonas*, *Paracoccus*, *Stenotrophomonas*	*Oryzais melastigma*
Ye et al. [74]	No highlight or direct report of importance	*Aeromonas*	Treatment group: Increase of Proteobacteria, Acidobacteria, Gemmatimonadetes, Spirochaetae, *Aeromonas*, *Brevinema*, and *Gemmatimonas* Decrease of Fusobacteria, Firmicutes, Cyanobacteria, *Nocardioides*, *Sphingomonas* Control group: Significantly higher abundance of *Clostridium *sensu stricto* 1, Mycobacterium,* and* Blvii28 wastewater sludge groups*	*Hypophthalmichthys nobilis*
Medriano & Bae [43]	No highlight or direct report of importance	*Cetobacterium*	Treatment groups: Increase of Fusobacteria, Proteobacteria, *Plesiomonas*, *Vibrio*, *Pseudomonas*, *Leucobacteria*, *Cetobacterium*, *Acinetobacter*, and *Shinella* Decrease of Firmicutes, Bacteroidetes, Actinobacteriota, *Chloroflexi*, *Crenarchaeota*, *Enterococcus*, *Enterobacter*, and *Kouleothrix*	*Danio rerio*
Lu et al. [39]	No highlight or direct report of importance	No highlight or direct report of importance	Treatment groups: Increase of Fusobacteria and Firmicutes Decrease of Verrucomirobiota Significant in *Simkania* and *Mucispirillum* Control groups: Significant in *Parachlamydia*, and *Candidatus_Xiphinematobacter*	*Oreochromis niloticus*
Jia et al. [25]	*Acinetobacter*, *Bosea*, and *Shewanella*	*Lactobacillus*	Treatment groups: Increase of *Acinetobacter*, *Bosea*, *Bacteroides*, *ZOR006*, and* Shewanella* Decrease of *Lactobacillus* and *Escherichia-Shigella*	*Ctenopharyngodon idella*
Hou et al. [24]	*Vibrio*	No highlight or direct report of importance	Treatment group: Increase of Proteobacteria, *Hyphomicrobium*, and *Legionella,* and *Vibrio* (after 28 days) Decrease of Actinobacteriota, *Mycobacterium*, *Sphingomonas*, *Defluviimonas*, *Hyphomicrobium* (after 28 days), *Mycobacterium* (after 28 days), and *Reyranella*	*Gobiocypris rarus*
Chen et al. [13]	No highlight or direct report of importance	*Cetobacterium*	Treatment Group: Increase of Actinobacteriota, Proteobacteria, Fusobacteriota*, Cetobacterium*	*Cyprinus carpio L*
Usman et al. [64]	*Aeromonas* and *Ralstonia*	No highlight or direct report of importance	Treatment group: Increase of *Aeromonas*. Also, unique marker for MP-HIGH Unique marker for MP-LOW; *Ralstonia*, *Paraburkholderia*, *Pelmonas*, *Staphylococcus*, *Bradyrhizobium*, and *Pararhizobium*	*Oryzias javanicus*
Yanyun Liu et al. [37]	No highlight or direct report of importance	*Butryricicoccus* and *Faecalibacterium*	Treatment group: Increase of *Butyricicoccus* and *Eysipelotrichaceae UCG-006* Decrease of *Faecalibacterium* and *Filifactor*	*Dicentrarchus labrax*
Guo et al. [22]	*Bosea*, *Mycoplasma*	*Shewanella*, *Gordonia*, and *Pseudomonas*	Treatment group: Increase of Actinobacteriota, *Methylobacterium*, *Microbacterium, Rikenellaceae RC9 gut group, Neochlamydia Candidatus, Protochlamydia*, *Bosea, Bradyrhizobium, Mycoplasma*, *Gordonia*, and *Pseudomonas* Decrease of Proteobacteria, Firmicutes, *Edwardsiella*, and *Shewanella*	*Danio rerio*
Zhao et al. [80]	*Aeromonas*, *Shewanella*, and *Pseudomonas*	No highlight or direct report of importance	Treatment group: Increase of Proteobacteria, Fusobacteria, *Chloroflexi*, *Aeromonas*, *Shewanella*, *Microbacterium*, *Nevskia*, and* Methyloversatilis* Decrease of Firmicutes, Actinobacteriota, Acidobacteria, Gemmatimonadetes, Cyanobacteria, *Bacteroides*, *Pseudomonas*, *Ralstonia*, *Stenotrophomonas*, *Chryseobacterium*, *Rhizobiaceae*, *Sphingomonas*, *Variovorax*, *Rhodococcus*, *Roseburia*, *Butyrivibrio*, *Lysobacter*, *Phascolarctobacterium*, *Mycobacterium*, *Micromonospora*, and* Gaiella*	*Danio rerio*
Ouyang et al. [47]	*Flavobacterium*, *Shewanella*, and *Plesiomonas*	No highlight or direct report of importance	Treatment group: Increase of Bacteroidetes, *Flavobacterium*, *Shewanella*, *Flavobacterium*, unclassified *Enterobacteriaceae*, *Proteocatella*, *Plesiomonas*, unclassified *Barnesiellaceae* and* Cetobacterium* Decrease of Firmicutes, *Enterococcus*, and *Nocardia*	*Cyprinus carpio*

At the genus level, several different bacteria populated the gut microbiome of fish, their abundances, and presence varying across each study. Among those studies popular genera appearing in multiple studies included *Vibrio*, *Pseudomonas*, *Lactobacillus*, *Aeromonas*, *Shewanella*, and *Cetobacterium*.

*Vibrio* was observed to show an increased abundance in treatment groups across all its mentioned studies, whereas *Lactobacillus* was shown to decrease in study groups except for Zhou et al. [81] and Zhang et al. [78]. Both *Aeromonas* and *Pseudomonas* were observed to increase within study groups except for Zhu et al. [83], with the latter also decreased within studies by Fang et al. [21] and Zhou et al. [81]. Similarly, *Shewanella* was abundant within treatment groups except for Guo et al. [22]. *Cetobacterium*, on the other hand, was observed to overall increase in most study groups except for Byeon et al. [11], Chen et al. [14], and Bao et al. [9]. Interestingly Bao et al. [9] demonstrated that *Cetobacterium* both increased and decreased within their respective treatment groups in their study.

**Fig. 4:**
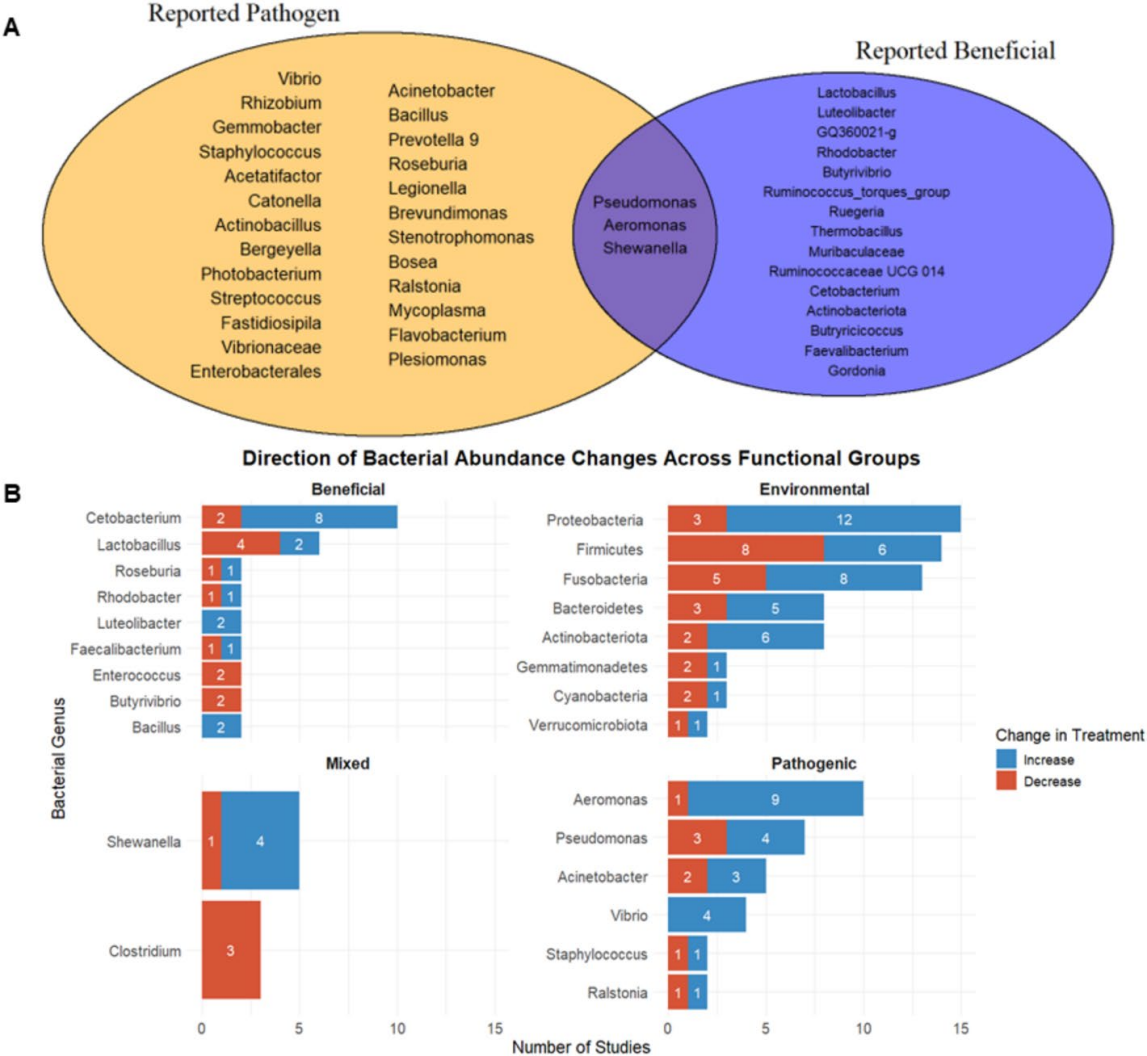
**A** Venn diagram of bacterial genera identified as beneficial or pathogenic across reviewed studies; **B** frequency of studies reporting directional changes for abundance of key bacteria, faceted by functional role

The studies also listed bacteria which were found to be pathogenic and beneficial to their respective studies (Fig. 4A). These observations were summarized into trends to showcase the direction of bacterial abundance changes of the most frequently reported findings (Fig. 4B). The pathogens were *Vibrio*, *Pseudomonas*, *Aeromonas*, *Rhizobium*, *Gemmobacter*, *Staphylococcus*, *Acetatifactor*, *Catonella*, *Actinobacillus*, *Bergeyella*, *Photobacterium*, *Streptococcus*, *Fasidiosipila*, Vibrionaceae, Enterobacterales, *Acinetobacter*, *Bacillus*, *Prevotella 9*, *Roseburia*, *Legionella*, *Brevundimonas*, *Stenotrophomonas*, *Bosea*, *Shewanella*, *Ralstonia*, *Mycoplasma*, *Flavobacterium*, and *Plesiomonas*. As for beneficial bacteria, the studies listed *Lactobacillus*, *Luteolibacter*, *GQ360021-g*, *Rhodobacter*, *Butyrivibrio*, *Ruminococcus_torques_group*, *Shewanella*, *Ruegeria*, *Thermobacillus*, *Pseudomonas*, *Aeromonas*, *Muribaculaceae*, Ruminococcaceae UGG 014, *Cetobacterium*, *Actinobacteriota*, *Butryricicoccus*, *Faevalibacterium*, and *Gordonia*. Interestingly there were conflicting findings for *Aeromonas*, *Pseudomonas*, and *Shewanella* where Byeon et al. [11], Zhu et al. [83], Fang et al. [21], Ye et al. [74], and Guo et al. [22] reported these bacteria to be beneficial in their studies.

## Discussion

In this review, all studies implemented the use of the Illumina sequencing platform to conduct their sequencing except for Liu et al. [36, 38] which employed ONT systems (Table 1). In past instances, the reliability of reads from ONT systems was concerning due its high error rate [32],however with recent improvements to its base calling algorithm, their capabilities are comparable and, in some cases, better than those found in Illumina when dealing with amplicon reads [28]. Additionally, Liu and colleagues also used ONT systems to perform metagenomic sequencing which targets the entirety of DNA and not just a specific region, eliminating the need to use specific primers [59]. This also allows more information to be gained from the raw DNA reads.

Alpha-diversity can be defined as species diversity that would be observed within a focused habitat or location [2]. It is normally measured via two considerations: species richness, which accounts for the total number of different species within a selected location, and species evenness, which considers the evenness of the distribution for the differing species within a community, often revealing the more dominant feature [69]. All studies within this review implored the usage of metrics that accounted for both the species richness and evenness expected in alpha-diversity analysis (Table 3). Beta-diversity instead measures the differences or variation of species composition between areas or communities [55]. While the use of diversity metric for the beta-diversity analysis was not completely ideal, their usage of PCA and similar plots more than make up for their lacking usage of certain metrics (Table 3). These studies were still able to portray a difference in microbial composition between study groups while also employing reliable statistical evidence, allowing these findings to be vouched. Both alpha- and beta-diversity analysis [24] showed that the gut microbiome somewhat plays a role in the studies included for this review.

Multiple bacteria were showcased to have fluctuated within treatment groups compared against their respective control groups, as shown in Figs. 3 and 4. These bacteria shift patterns could also be categorized in accordance with their proposed functional group toward the study, mainly as an overall benefit, pathogen, mixed, or stable environmental presence toward the host. While their major observed fluctuations can be telling for a bacteria’s immediate role and effect toward the host, context is required to better understand how the said shifts contribute to overall health of the host.

At the phylum level, Proteobacteria, Firmicutes, Bacteroidetes, Actinobacteriota, and Fusobacteria mainly comprised the gut microbiome of fish with some occasional appearances from bacteria such as Gemmatimonadetes, Cyanobacteria, and Verrucomicrobiota. These patterns are recorded in Table 4 and summarized in Fig. 4. Other popular bacteria found within fish include the genera of *Vibrio*, *Pseudomonas*, *Lactobacillus*, *Aeromonas*, *Shewanella*, and *Cetobacterium*. The abundance of these features often shifted across studies with multiple bacteria showing conflicting findings between said studies. While the levels of each bacterium within the gut are not conclusive in nature, their sudden shifts in abundances between control and treatment groups are vital. These shifts in abundances and composition are referred to as a state of dysbiosis [60], often being attributed to multiple health maladies like IBD [41], cancer [1], and obesity [15]. Additionally, gut microbiome dysbiosis is quite common in those suffering from ailments as well. Gut microbiota play a vital role in health whether it be downregulating cholesterol production [29], facilitating protein or amino acids digestion and absorption [3], as well as providing resistance against pathogens and regulating immune responses [24]. This dysbiosis is harmful as it signifies the gut being out of equilibrium, effectively losing its ability to maintain the host’s health.

Different bacteria play different roles within the host. Bacterial members under the phylum Proteobacteria have primarily been shown to be involved in nitrogen degradation as well as complexation of carbons [82]. Other members have also shown the capability to induce inflammation and harm the intestinal lining [27]. Variations to its abundance act as indicators of fish intestinal microbiota alteration within the host [21]. A reduced presence can affect the host’s ability to regulate metabolic digestion [78]. On the other hand, a spike in abundance may lead to the production of lipopolysaccharides that can cause inflammation, disruption of the intestinal mucosal barrier, and increased permeability of intestinal walls [77]. Similarly, higher presences of Proteobacteria often correlate with sicker individuals [79]. Trends would indicate that Proteobacteria in moderate levels signify a healthy host, whereas a spike in abundance would suggest a sicklier host. Additionally, the phylum Actinobacteriota, one of the larger bacterial phyla, represents an excellent collaborator in producing pharmaceutical products such as antimicrobial agents, antibiotics, and industrial enzymes [50]. The phylum is important given its role to synthesize secondary antibiotic metabolites toward aggressive pathogens, its reduction potentially leads to the weakening of the intestinal barrier and an increased sensitivity to immune stimulus [47]. Bacterial members also function to enhance disease resistance in fish and promote the development of immune organs. Conversely, studies also show a reduction in abundance may lead to digestive tract diseases within the host [58]. Given its association with more healthier benefits, higher-level Actinobacteriota is preferred as it may aid in host’s ability to better regulate their overall health, especially within more challenging living conditions.

Furthermore, another large component of the gut microbiome in fish includes the phylum Firmicutes. Mostly known for promoting host growth, these bacterial members aid in promoting the uptake of nutrients [20] as well as influence the metabolism of glucose, lipids, and energy of the host [72, 73]. They are major producers of butyrate [65] and help to provide nutrition toward the mucosal cells within the intestines which maintains the normal function and regulates the intestinal micro-ecological environment [23]. They are also major participators of fatty-acid absorption [75]. Its reduction may inhibit nutrient absorption [47] and has been marked as a key signature of IBD-like colitis in certain fishes [7], although an abundance of the bacteria has also been reported to be linked to the development of obesity [83]. Overall, the phylum’s presence within a host is an overall benefit. In most cases where Firmicutes is observed to decrease in numbers, it is often linked to a dysbiosis state or complications [60]. The phylum Bacteroidetes is another common member of the intestinal microbiome. It plays a crucial purpose in maintaining the dynamic balance of the mucosal immune system within the intestine [57]. It is also important for diet digestion due to its involvement in protein metabolism and carbohydrate transport [81]. While some members are pathogenic, there are also others that function to colonize the intestinal mucosa, functionally inhibiting the colonization of potential pathogens within the intestines [63]. Similarly, the phylum also showed associations with gene expression related to nutrient absorption, metabolism, and strengthening the mucosal barrier [83]. Trends suggest that Bacteroidetes larger presence is a beneficial function to the host but not as a sign of good health, instead acting as countermeasure to tackle complications the host might be facing. Another phylum found within the intestine of fish is Cyanobacteria, a feature that is ubiquitous across most ecosystems [17] and is a common food source for aquatic animals such as shrimp, zooplankton, filter feeders, and omnivorous fish [61]. Certain members have also been shown to possess the ability to produce microcystin toxins, which can impair the growth and development of aquatic life when exposed [51]. Furthermore, the phylum Verrucomicrobiota, which are mucin-degrading bacteria, were found in fish intestines as well; their presence believed to regulate gut health [12]. Studies also correlate their reduction with growth-rate decrease while elevation with increased body weight [39]. The presence of both these phyla could more function as an environmental presence to host, functioning either to suppress issues or act as an opportunistic pathogen given the proper environment. While bacterial phyla have an observable effect toward the health of their respective hosts, their presence and fluctuations are more of an indicator toward the health status for the host instead of direct factor that directly affects them.

In terms of genus, *Vibrio* is commonly found within fish where they function mostly as a pathogen which poses health risks to both humans and aquatic life [6]. The bacteria are known to cause enteritis, which can lead to cholera and sepsis among humans [19] or inhibit the pathways associated with nutrient metabolism and absorption, as well as signal transduction [30]. As a common pathogen, it also can cause serious economic losses in the fishing industry such as carps [70]. Another known pathogen found in fish was *Pseudomonas*. While these bacteria have been shown to cause lung infections within humans [80], other studies point it to be beneficial to fish like those experiencing salt stress [35]. They have potential as probiotics, showing capabilities to secrete signals that promote the proliferation and renewal of intestinal epithelial cells [52]. Studies also showed that it can protect zebrafish from *Flavobacterium* infection which causes numerous columnar and bacterial gill diseases [56], whereas its reduction may also affect the morphological structure of the intestines [83]. Additionally, another known pathogen is *Acinetobacter*,their appearance is more prominent within stressful environments [21]. Other studies have also linked it to be resistant toward antibiotics, further contributing toward the growing antibiotic-resistance issue [81]. Moreover, *Aeromonas* were also commonly found within the gut of fish. They are common inhabitants of estuary and freshwater environments with pathogenic tendencies affecting both fish [14] and humans [8]. The bacteria can cause severe economic losses by incurring motile Aeromonas septicaemia with high mortality or intestinal inflammation and soft tissue infection [54]. Other studies have also pointed out the commensal function of the bacteria within fish intestines [80]. These bacteria also possess the ability to secrete signals which promote the renewal and proliferation of intestinal epithelial cells [83]. Some species can even produce cellulase [74], the cellulolytic activity useful to compensate the deficiency of certain fish to derive their energy and nutrients from such a source [33]. Studies also showed that a decreased abundance of the bacteria can results in a change toward the morphological structure of the intestines [83]. Additionally, *Shewanella* was another bacterium found among fish. It is an opportunistic pathogen that can infect various aquatic life [48, 71], causing localized legions like keratoconjunctivitis and dermatitis. However, studies also highlighted its benefits, being associated with plastic degradation of materials like low-density polyethylene and polycaprolactone [26], as well preventing immune responses brought up by other gut bacteria [73].

Other notable genera found within fish gut include *Lactobacillus*. The bacteria are a known probiotic that promotes host growth by promoting the synthesis of digestive enzymes and minerals as well as regulating gut microbiota [5]. Furthermore, it has protective properties where it can secrete antibiotic substances against pathogens and produce lactic acid which can inhibit the growth of bacteria as well as enhance the antimicrobial activity of host lysozyme [78]. The bacteria have also been shown to be sensitive to oxidative stress [62], their presence is a potential response to intensive oxidative stress within the intestines. They also can remove heavy metals to a certain extent [36, 38]. Another bacterium within the fish intestine includes the *Cetobacterium*, a genus of specialized anaerobic bacteria commonly found within fish microbiota [43]. These microbes have great potential as probiotics [31]. They are able to produce vitamin B12, which contributes to overall fish nutrition [53], promote insulin expression which lowers blood glucose in fish [66], and create antimicrobial metabolites that can improve the immune system of the fish [43].

The role of bacteria varies from one another, some offering benefits to the host while others prove harmful. However, within this review a few notable features were found to have conflicting findings as *Shewanella* as well as *Pseudomonas* and *Aeromonas*, which were primarily identified as pathogens, were beneficial in some studies. While primarily known to be pathogenic to most organisms [14], these bacteria also possess beneficial traits that can prove useful in certain scenarios [26]. Context is crucial in understanding the roles of such bacteria toward their host,a major reduction or spike can turn any neutral commensal bacteria into a lacking beneficial feature or increase pathogenic problems accordingly. This links back to the previous statement about dysbiosis, an equilibrium gut is best for host health; major changes to any bacteria can prove to be detrimental to said host.

In summary, while pattern shifts of bacterial phyla do indicate the health of their host, their fluctuations are not directly that apparent to the role they play within the host. With the phyla Actinobacteriota and Bacteroidetes, for example, their increased presence could be signal of poor host health, their spike functioning more as the host’s natural response to the current situation. Firmicutes are perceived as good bacteria, and their decreased abundance can suggest sick hosts. Conversely, rising levels of Proteobacteria often correlate with sicker hosts. There are also phyla like Cyanobacteria and Verrucomicrobiota whose purpose within the host are opportunistic, their recorded fluctuations changing according to unique circumstances observed within the host. At the phylum level at least, these bacteria fluctuate in accordance with changes to host health and do not appear to be the primary driving force behind the decline of host health. The most frequent recorded pathogens were shown to be *Aeromonas*, *Pseudomonas*, *Acinetobacter*, and *Vibrio*, while notable beneficial bacteria were *Lactobacillus* and *Cetobacterium*. While the mechanism and roles of these bacterial genera were more apparent, others such as *Shewanella*, as well as both *Aeromonas*, and *Pseudomonas* have mixed functional roles within their respective host. These functional roles were more based on circumstances allowing them to function as both a pathogen and beneficial bacteria within the appropriate environment. Contextual understanding is important in considering the role of both potential beneficial bacteria and potential pathogens toward the fish gut microbiome in relation to health. In aquaculture environments, various fish species are kept in unique environments, making it challenging to establish a “universal” baseline for interpreting bacterial abundance in relation to fish health. Consequently, comparison between gut microbiome profiles of healthy control groups and experimental groups are essential for drawing meaningful conclusions. The validity of these comparisons can be extended to similar fish species or rearing conditions for more accurate inference.

Gut microbiome studies are inherently predictive, which introduces certain limitations. DNA-based sequencing captures all bacterial DNA; thus, there is no distinguishing between the DNA of non-viable and viable cells [40]. To address such limitations, further testing like cell culture plating, propidium monoazide (PMA) treatment [42], or RNA metagenomics [68] can be used to account for bacterial viability. Further research is needed to develop the gut microbiome analyses that are robust enough for health assessment without requiring additional scientific methods. The dynamic nature of the microbiome also limits the full insight that can be obtained based off single time-point sampling studies such as those showed in this review. These snapshots, while useful for initial insight toward microbial dynamics, do not fully explain the causal relationship bacteria may have with their host. To better account for this, future studies should adopt less invasive sampling methods and incorporate more longitudinal designs in their approach as to better assess the temporal shifts and stability of key bacterial taxa. Furthermore, another limitation of this review is that it is a systematic approach in analyzing the published results of each study and not a meta-analysis based on the reprocessed raw data made available from each respective study. Due to differences in each author’s use for sequencing platforms, primer sets, as well as approaches for bioinformatic pipelines, there might be potential biases introduced. For future meta-analysis-based studies, it is advisable to base them on publicly available raw data and uniformly process them using standardized parameters.

## Conclusion

In conclusion, the gut microbiome has an apparent effect toward fish health kept in aquaculture captivity. While this review cannot definitively give a universal baseline of gut microbiota usable across all types of fish, it can however provide the reader more contexts about the nuances of gut microbiome interaction in fish. Context is required to understand the role of each bacterium toward the gut health of fish, with different circumstances existing for different species. It also worth noting that larger sample sizes are quite important to validate the contribution of gut microbiota in fish health, a feat that is not often fully possible due to the invasive nature of sample collection for fish. Moving forward, the information listed in this review may prove useful for aspiring farmers especially when dealing with species that have never been domesticated or have little information recorded about them. Despite no concrete template of gut bacteria for domesticated fish, this review still can serve as future reference for readers on the general expectations when performing a gut microbiome study within aquaculture fish.

## Data Availability

All data generated or analyzed during this study are included in this article or can be made available by authors upon reasonable request.
